# Supplementary figures and images for: Characterization of four new monoclonal antibodies against the distal N-terminal region of PrPc
Source: PeerJ. 2015 Mar 19;3:e811. doi: 10.7717/peerj.811 (PMC4369333; doi:10.7717/peerj.811)

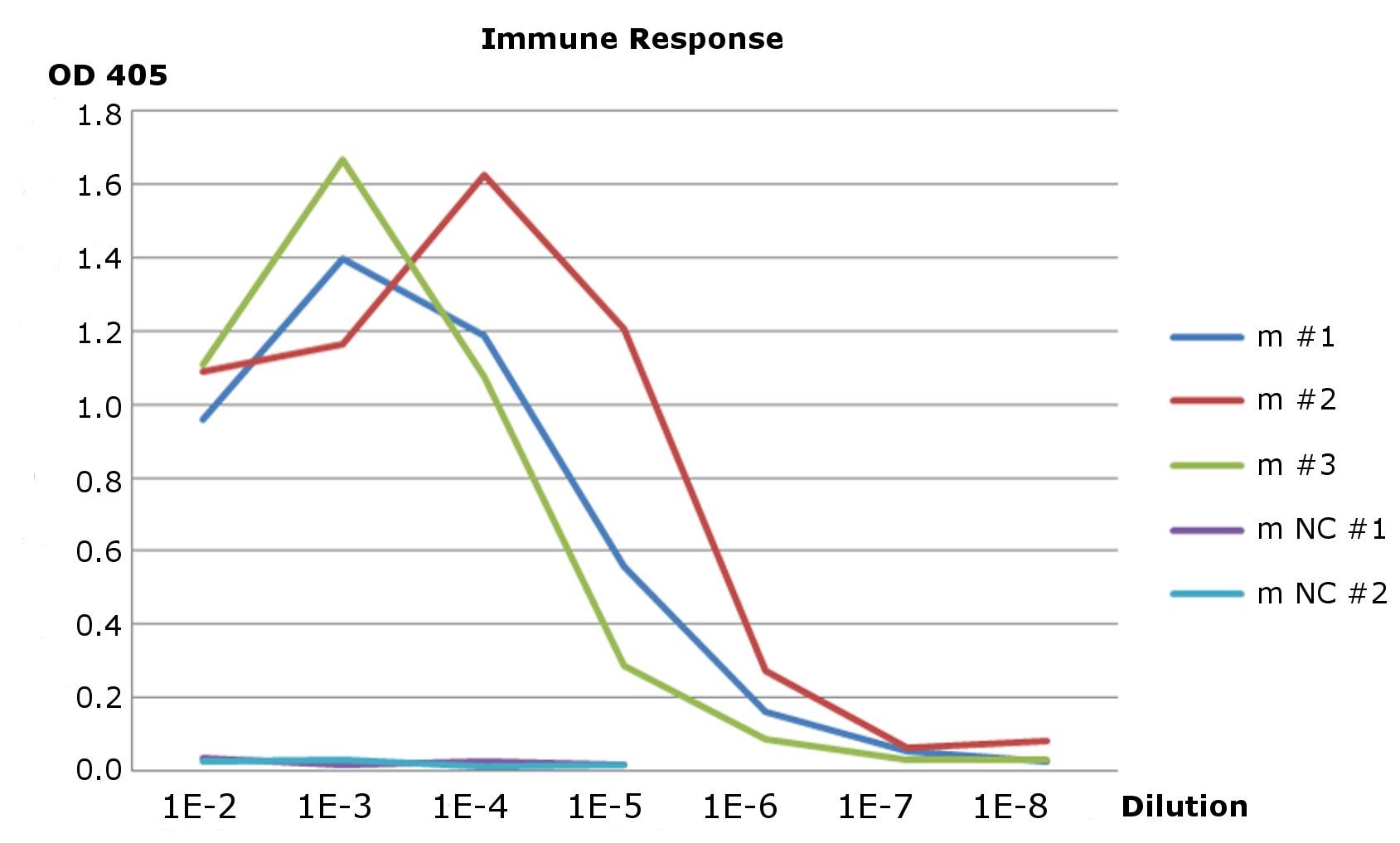

Supplement: Figure S1 — Immune sera from three mice and two negative controls were probed by ELISA against recombinant human PrP (recHuPrP). Serial dilutions were prepared for each sample. The sera from immunized mice were able to recognized recHuPrP up to the 10−6 dilution while both negative controls gave no reaction. [file peerj-03-811-s001.jpg]

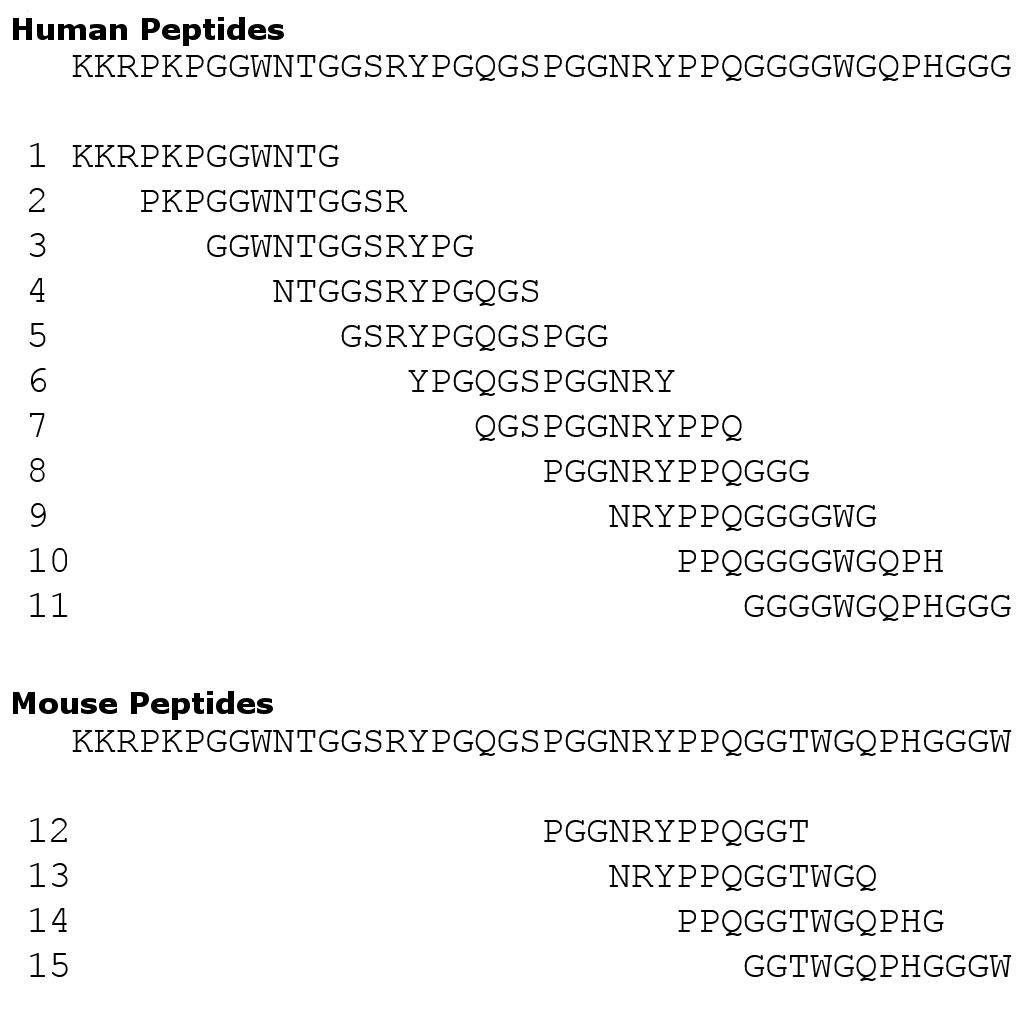

Supplement: Figure S2 — Panel of the synthesized peptides from HuPrP sequence (1–11) and MoPrP sequence (12–15) used for epitope mapping. [file peerj-03-811-s002.jpg]

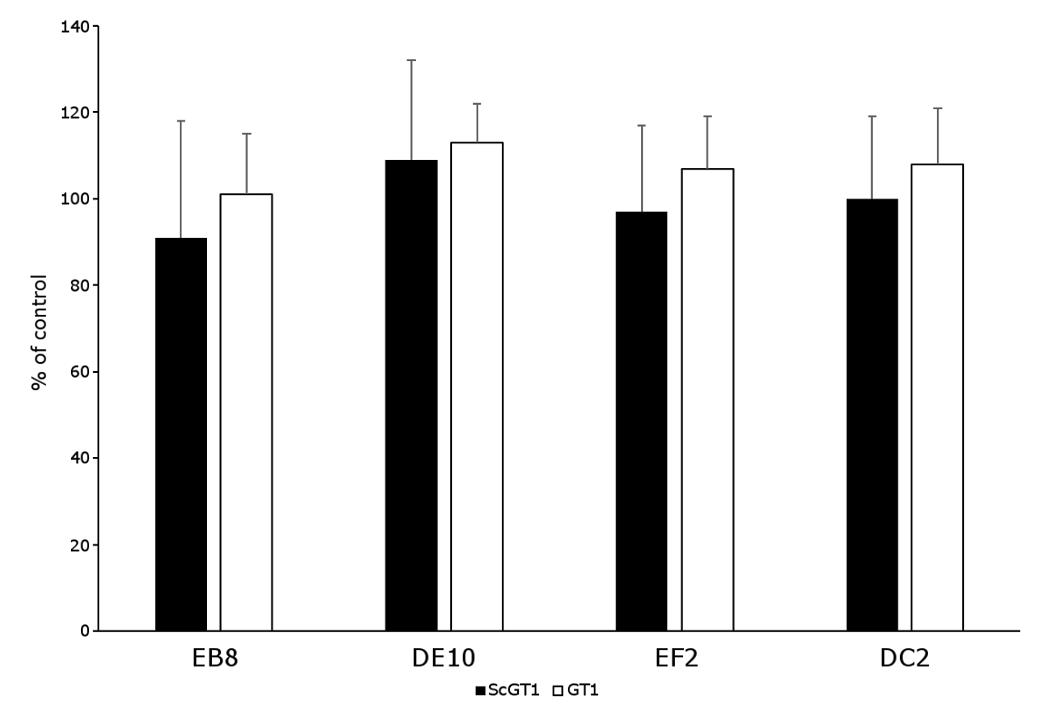

Supplement: Figure S3 — Both GT1 and ScGT1 cells were treated with the different mAbs for 5 days, refreshing the medium on the third day. Then cell viability was evaluated by MTT assay according to the procedure described in the ‘Materials and Methods’ section. No statistical differences in term of cell viability were found in mAb-treated cells compared to untreated controls. For every mAb the average values from 5 wells are expressed as percentages of cell viability referred to untreated cells. [file peerj-03-811-s003.jpg]
