# Supplementary material for: Characterization of four new monoclonal antibodies against the distal N-terminal region of PrPc
Source: PeerJ. 2015 Mar 19;3:e811. doi: 10.7717/peerj.811 (PMC4369333; doi:10.7717/peerj.811)

ELISA mapping

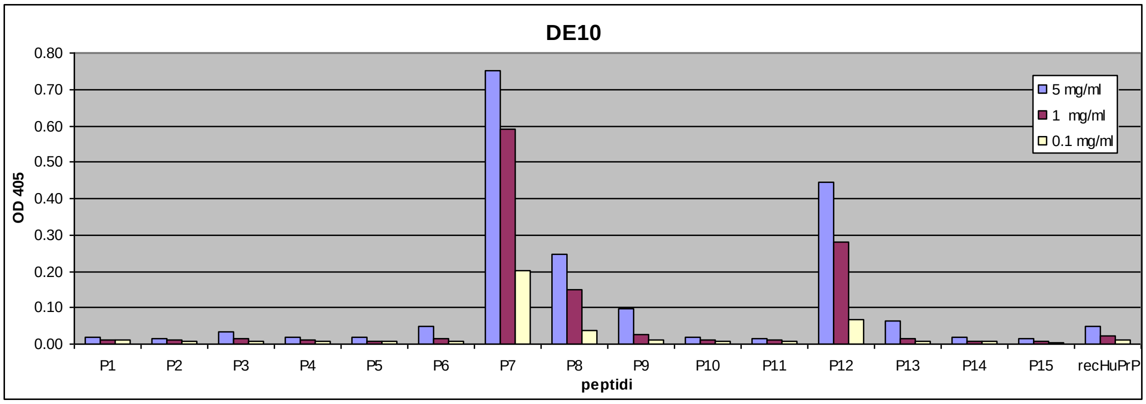

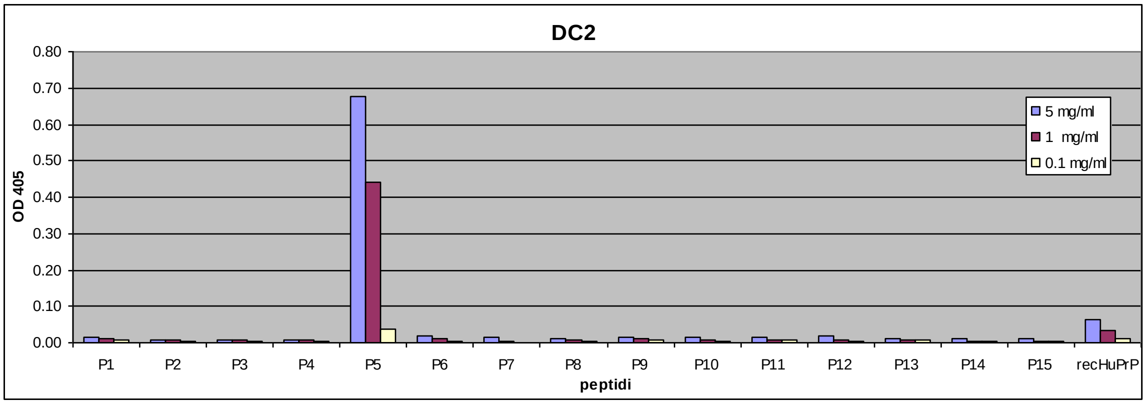

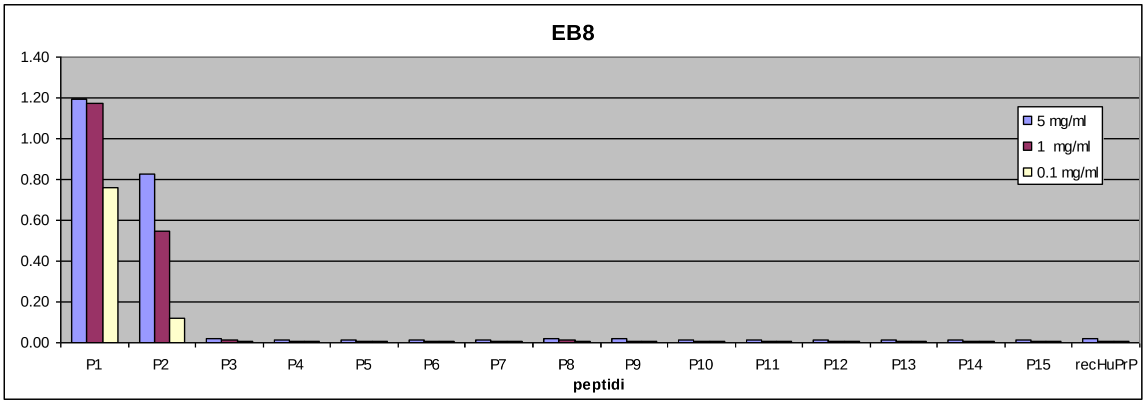

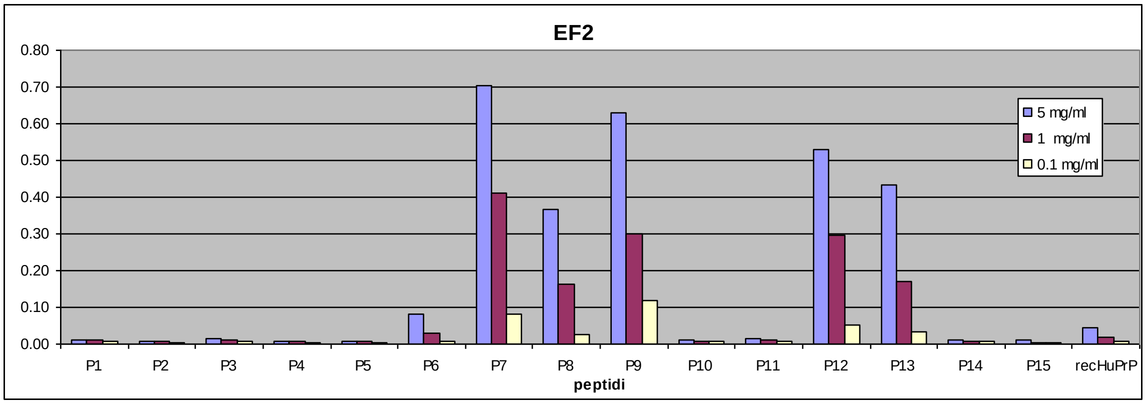

Western Blot analysis

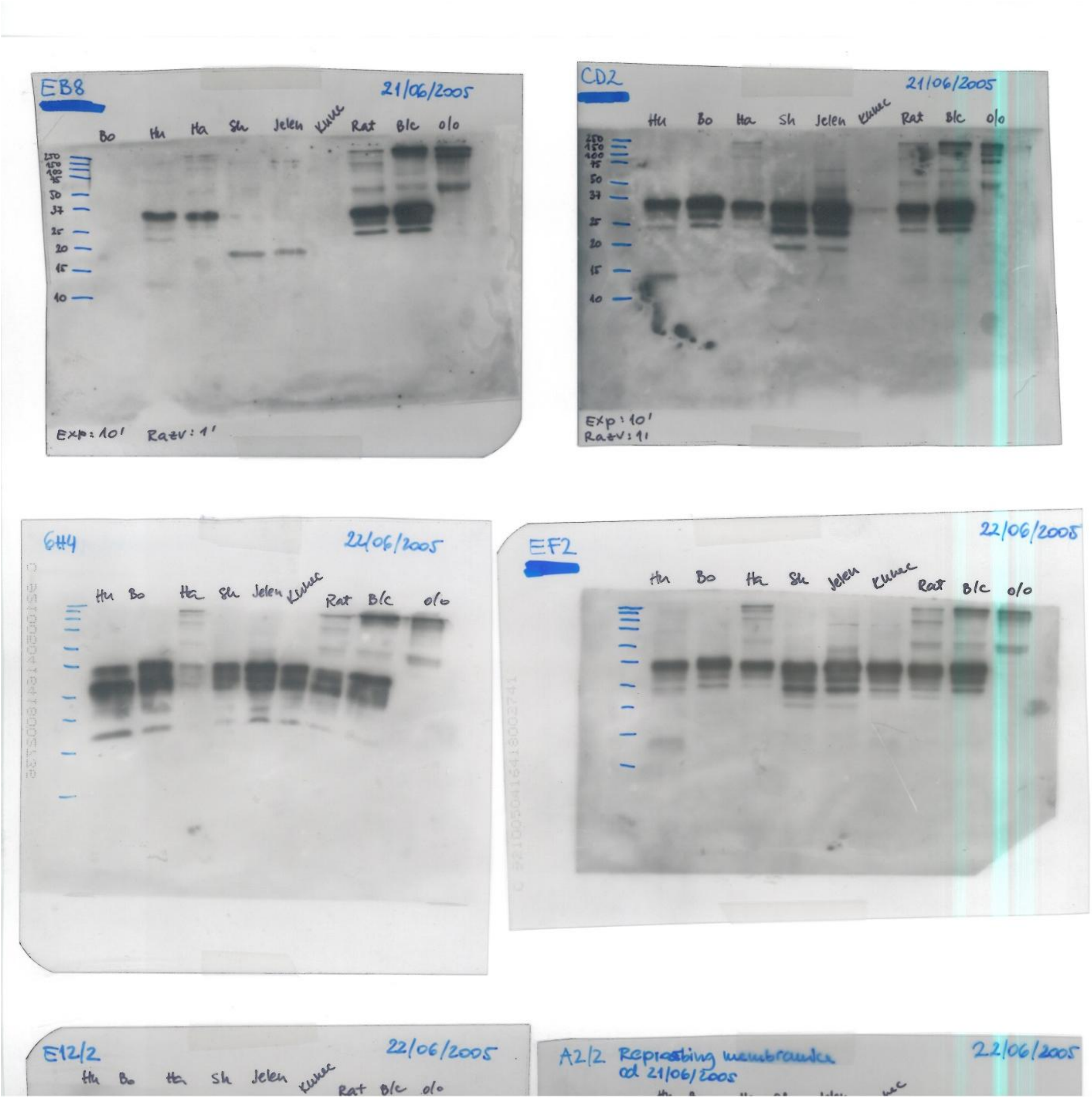

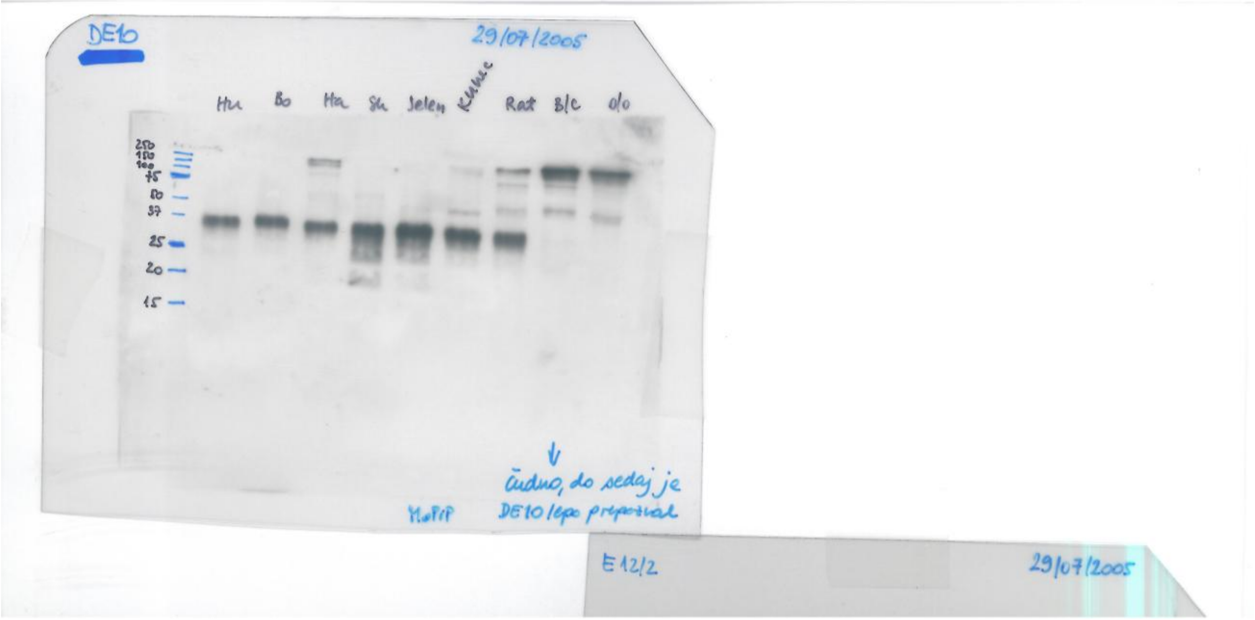
DE10
29/07/2005
Hu
Bo
Ha
Sh
Jelen
Rat
B/c
0/0
25
20
15
čudno, do sedaj je
DE10 lepo prepoznal
MoPrP
E12/2
29/07/2005

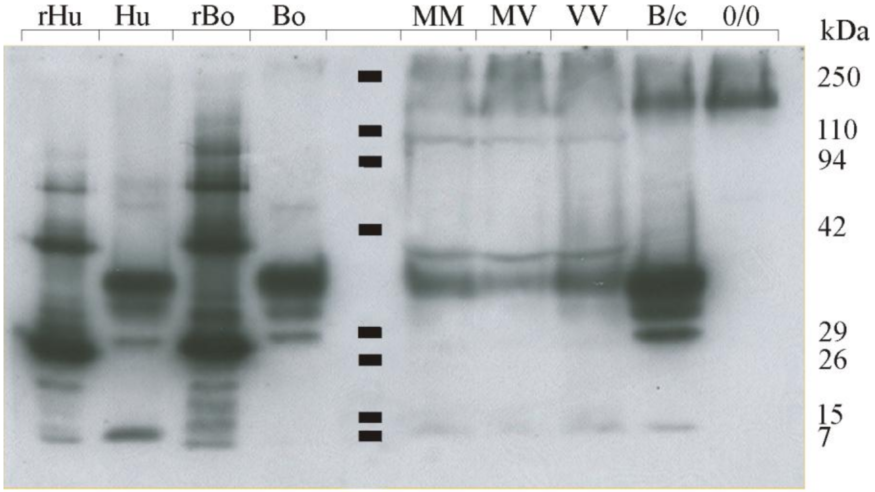
rHu
Hu
rBo
Bo
MM
MV
VV
B/c
0/0
kDa
250
110
94
42
29
26
15
7

DE10

GT1 cells stained with DE10 without Guanidium

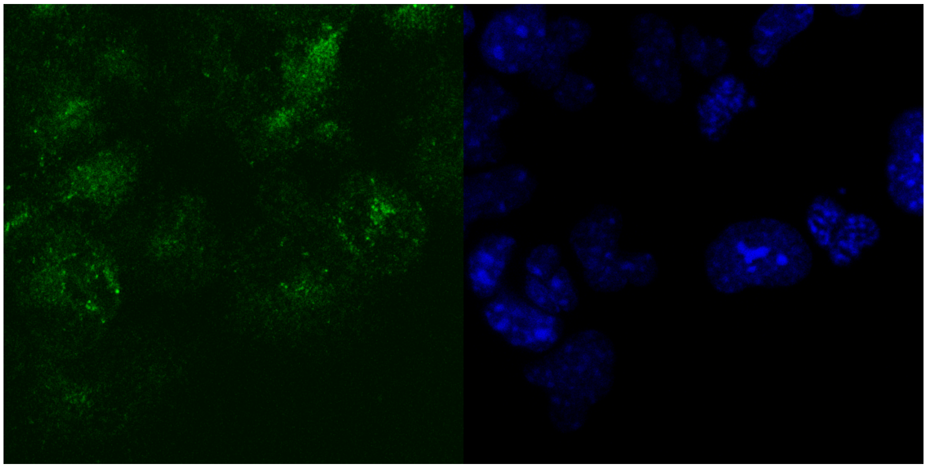

GT1 cells stained with DE10 with Guanidium

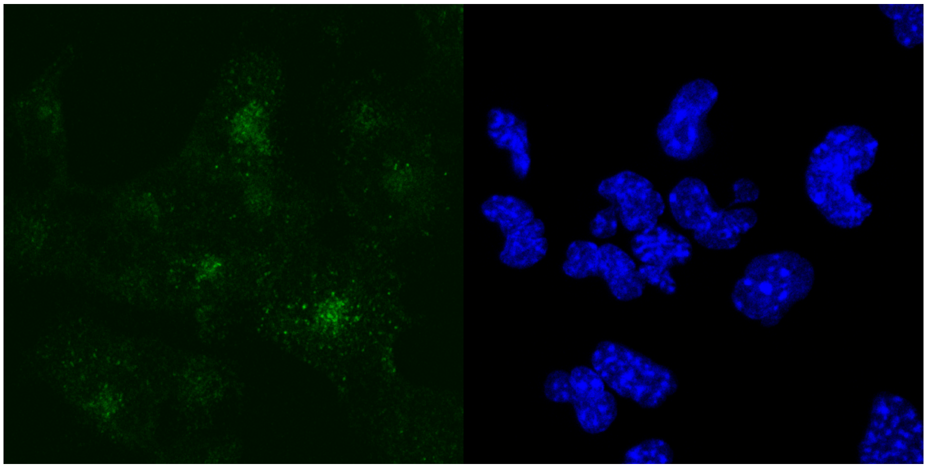

GT1 cells stained with DC2 without Guanidium

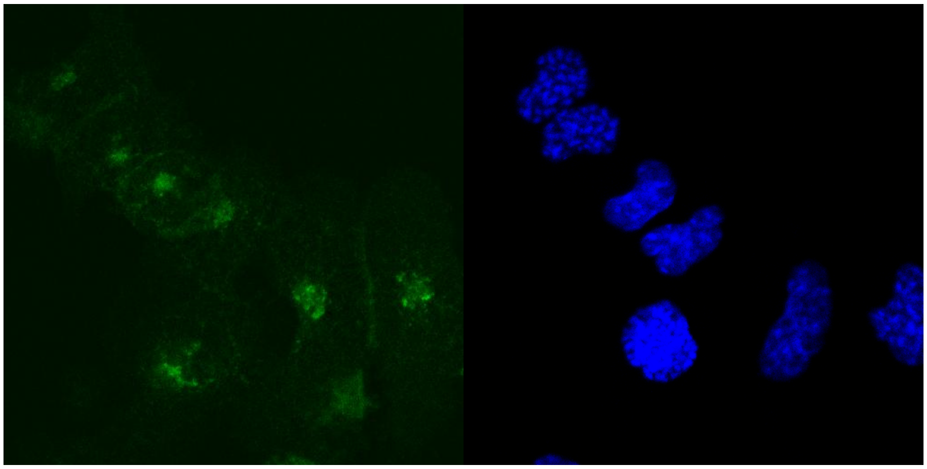

GT1 cells stained with DC2 with Guanidium

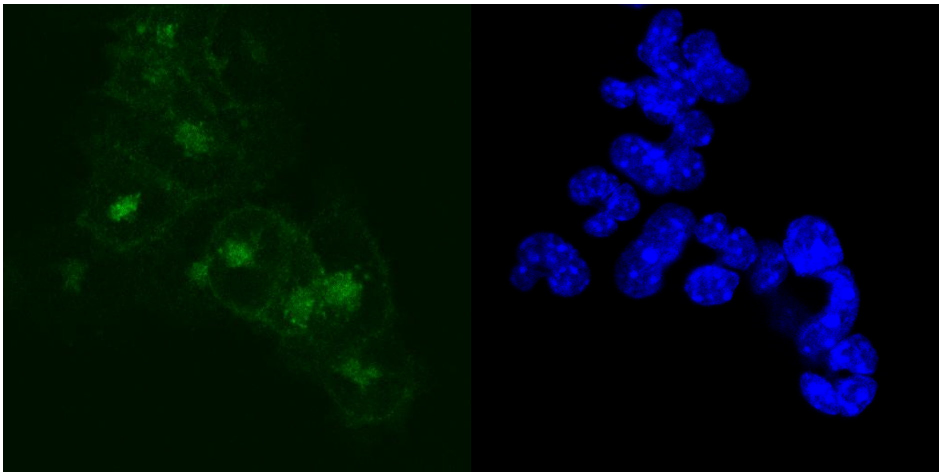

GT1 cells stained with EF2 without Guanidium

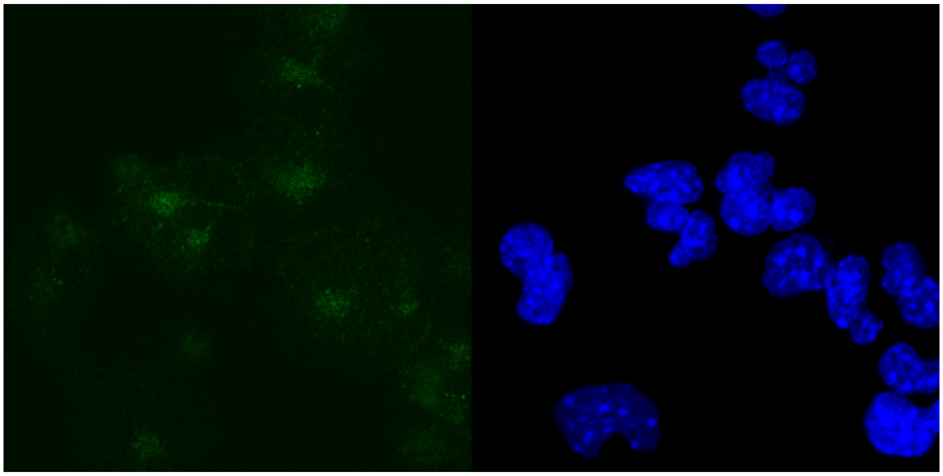

GT1 cells stained with EF2 with Guanidium

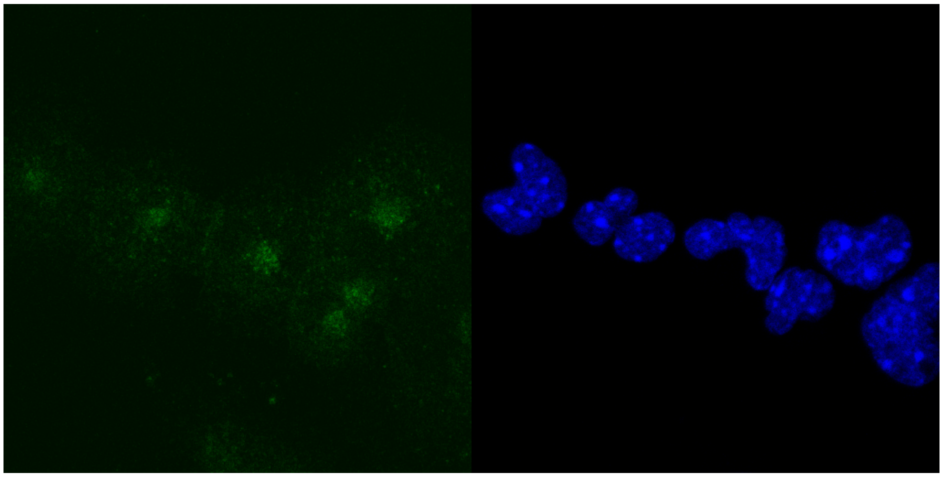

GT1 cells stained with EB8 without Guanidium

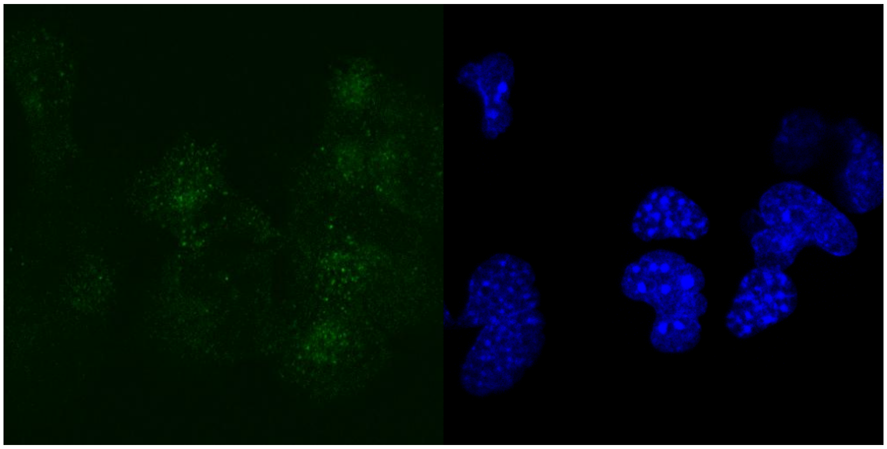

GT1 cells stained with EB8 with Guanidium

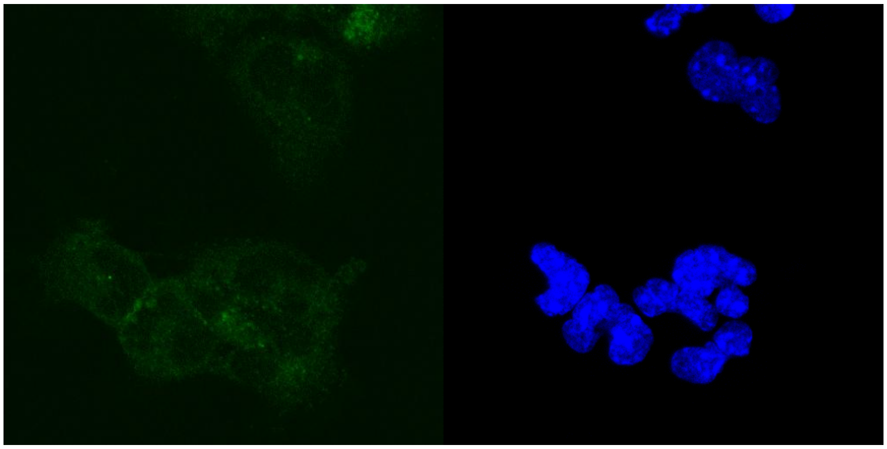

ScGT1 cells stained with DE10 without Guanidium

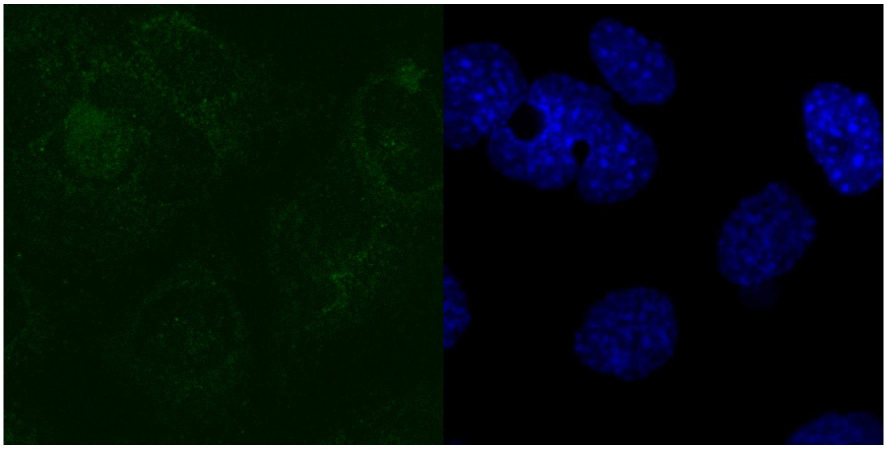

ScGT1 cells stained with DE10 with Guanidium

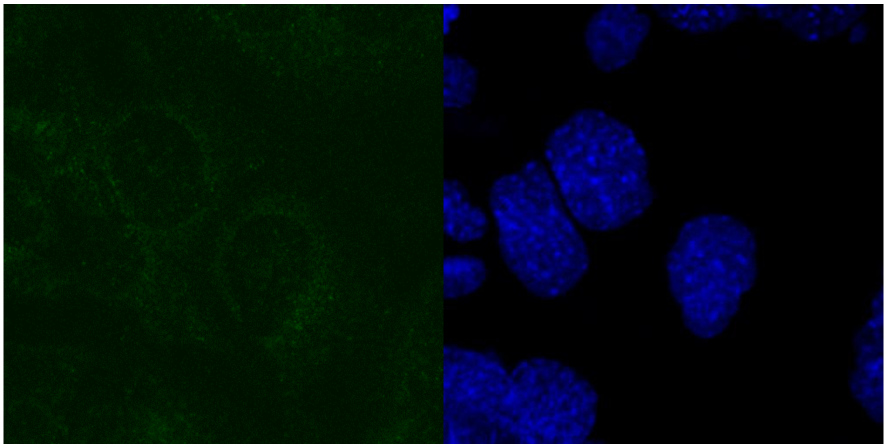

ScGT1 cells stained with DC2 without Guanidium

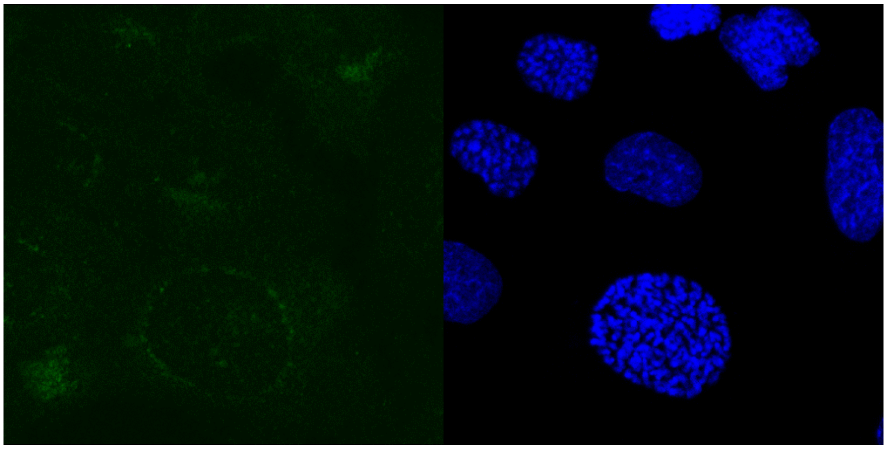

ScGT1 cells stained with DC2 withGuanidium

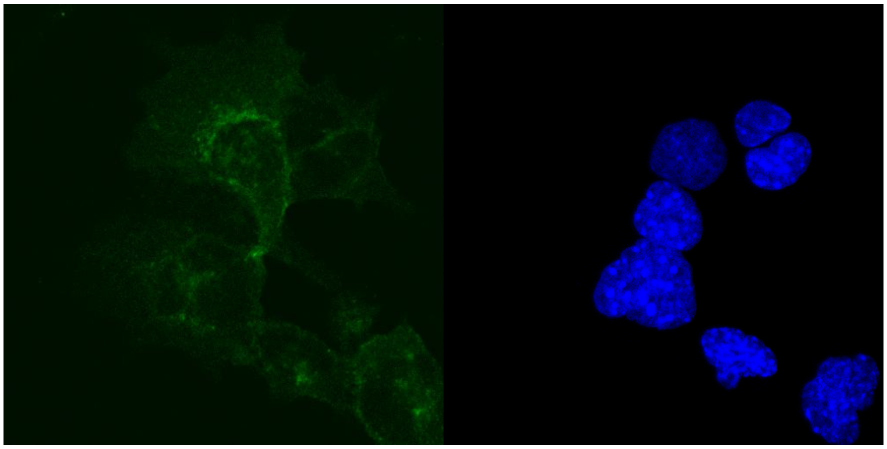

ScGT1 cells stained with EF2 without Guanidium

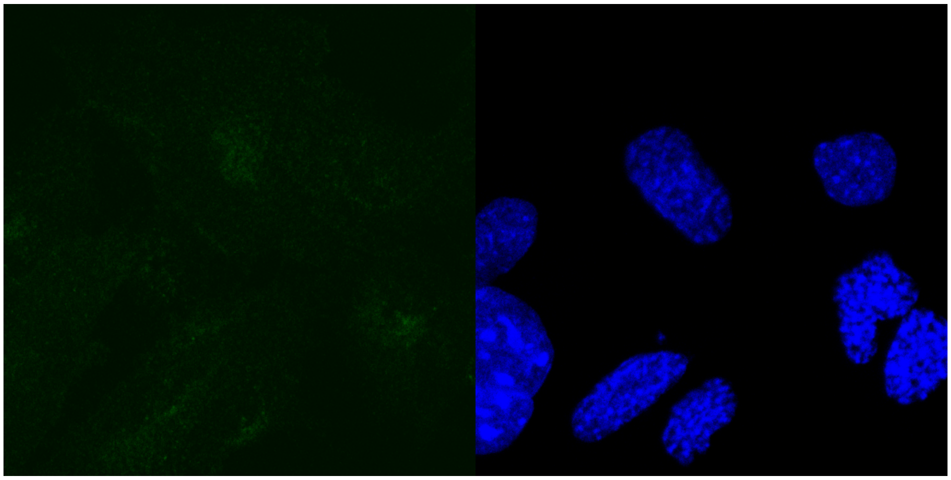

ScGT1 cells stained with EF2 with Guanidium

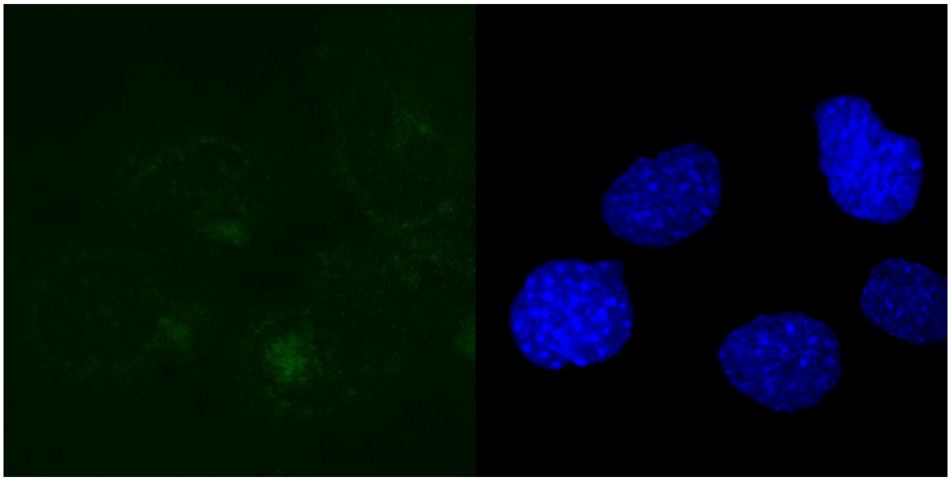

ScGT1 cells stained with EB8 without Guanidium

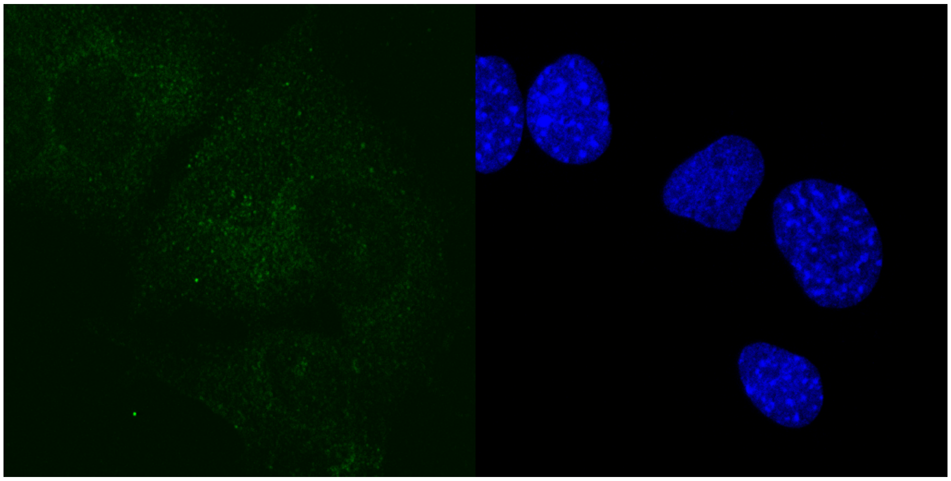

ScGT1 cells stained with EB8 with Guanidium

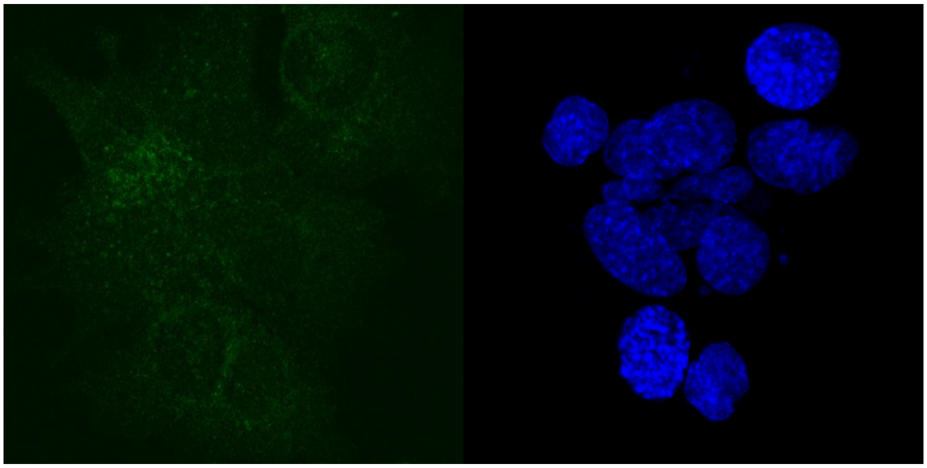

Total ERK blot in GT1 and ScGT1 treated with DE10

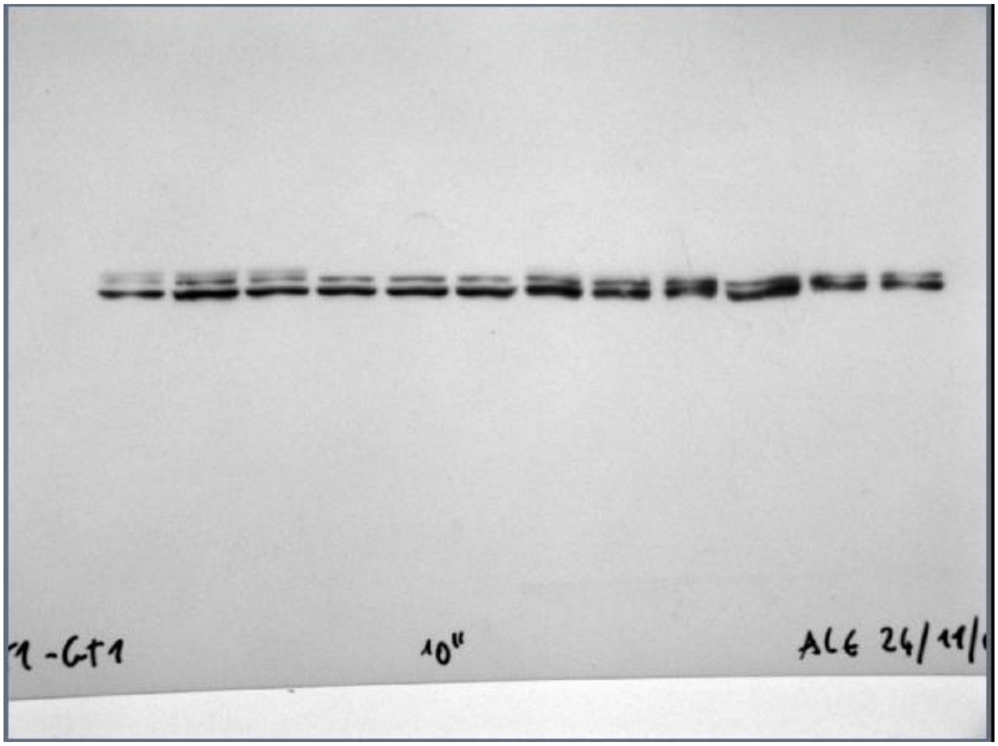

Phospho-ERK blot in GT1 and ScGT1 treated with DE10

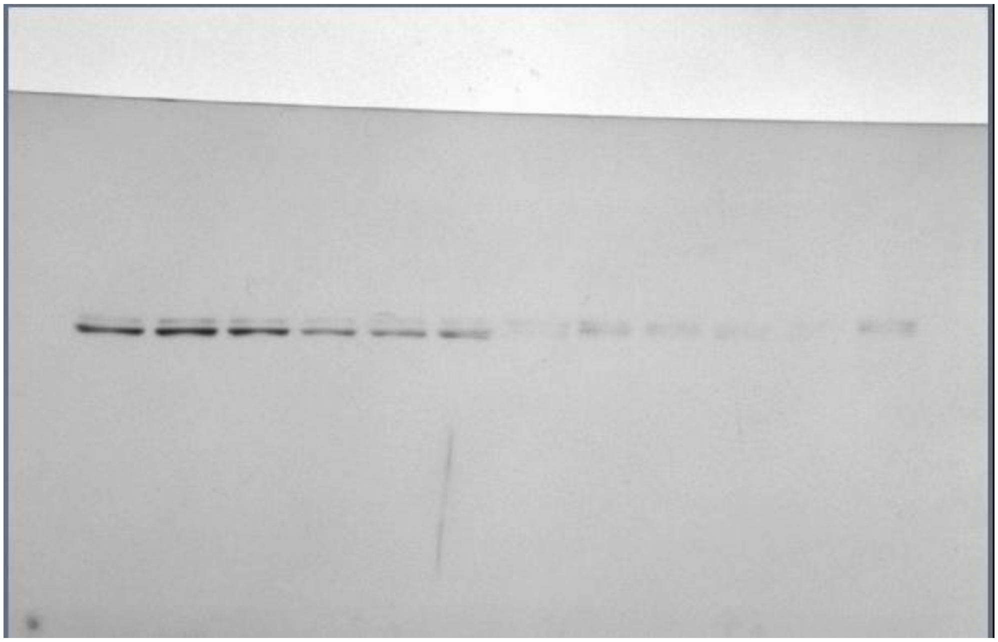

Total ERK blot in GT1 and ScGT1 treated with DC2

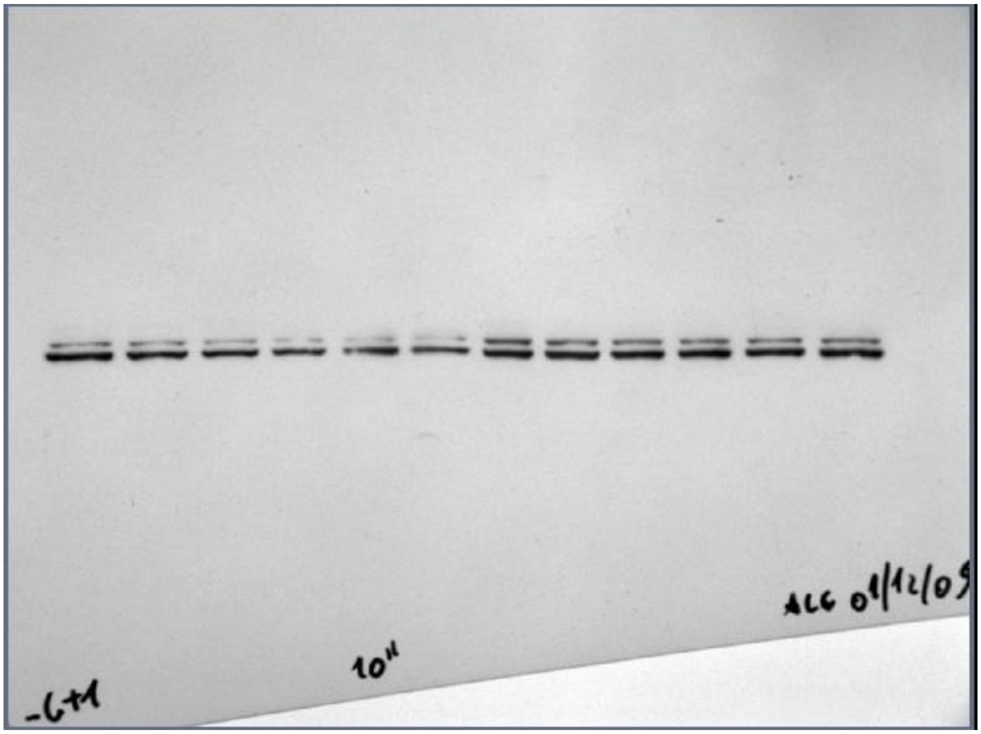

Phospho-ERK blot in GT1 and ScGT1 treated with DC2

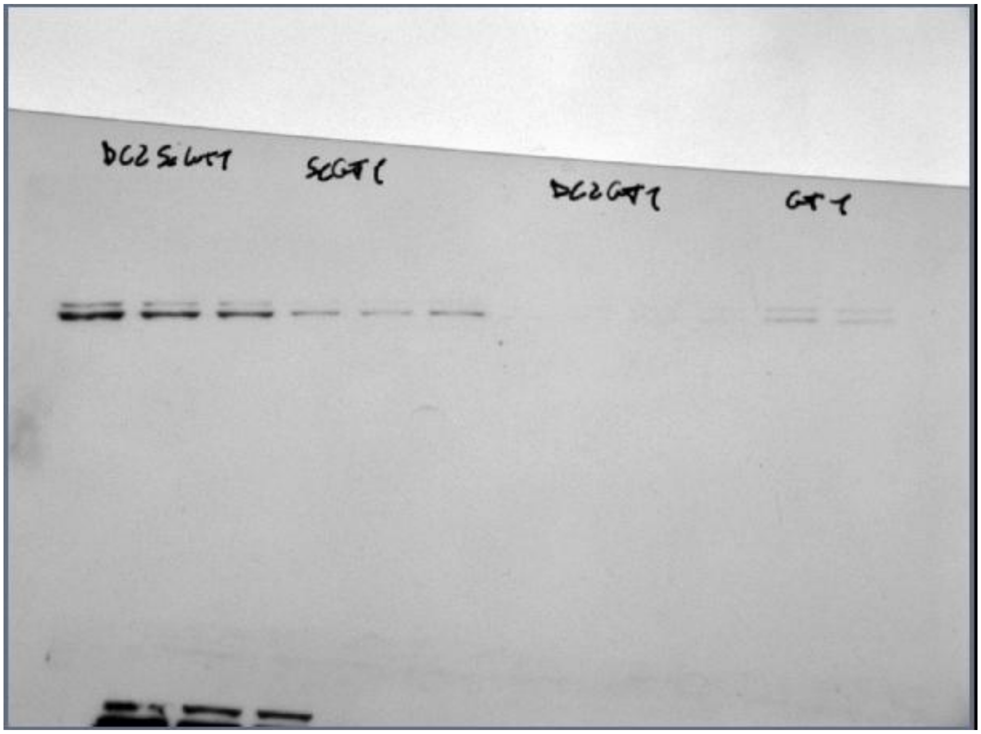

Total ERK blot in GT1 and ScGT1 treated with EF2

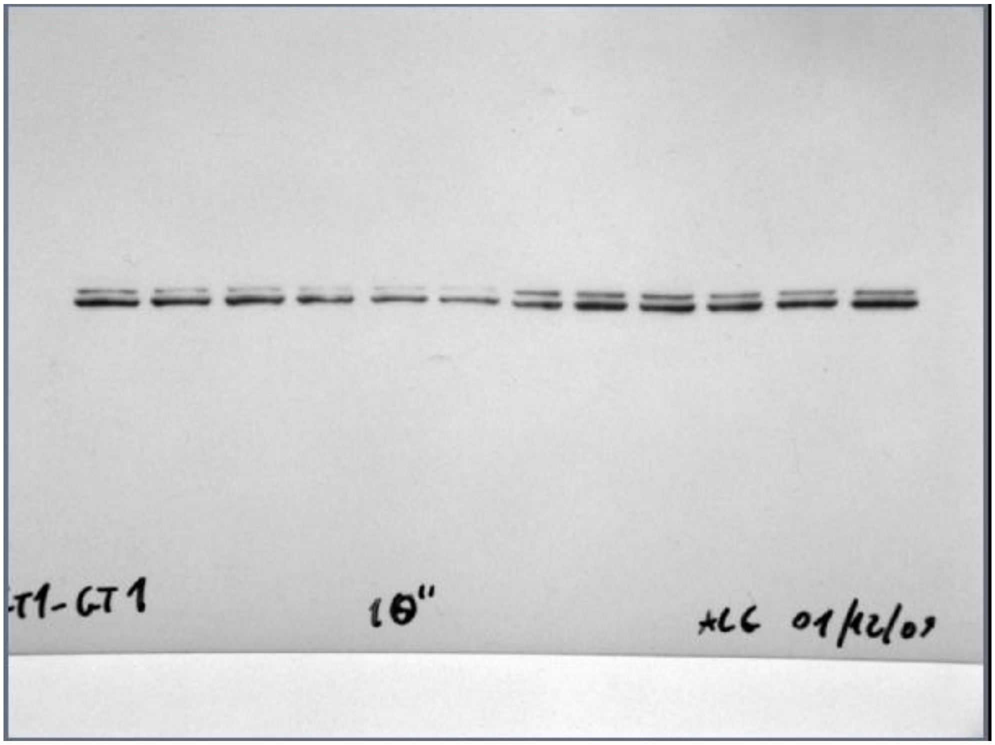

Phospho-ERK blot in GT1 and ScGT1 treated with EF2

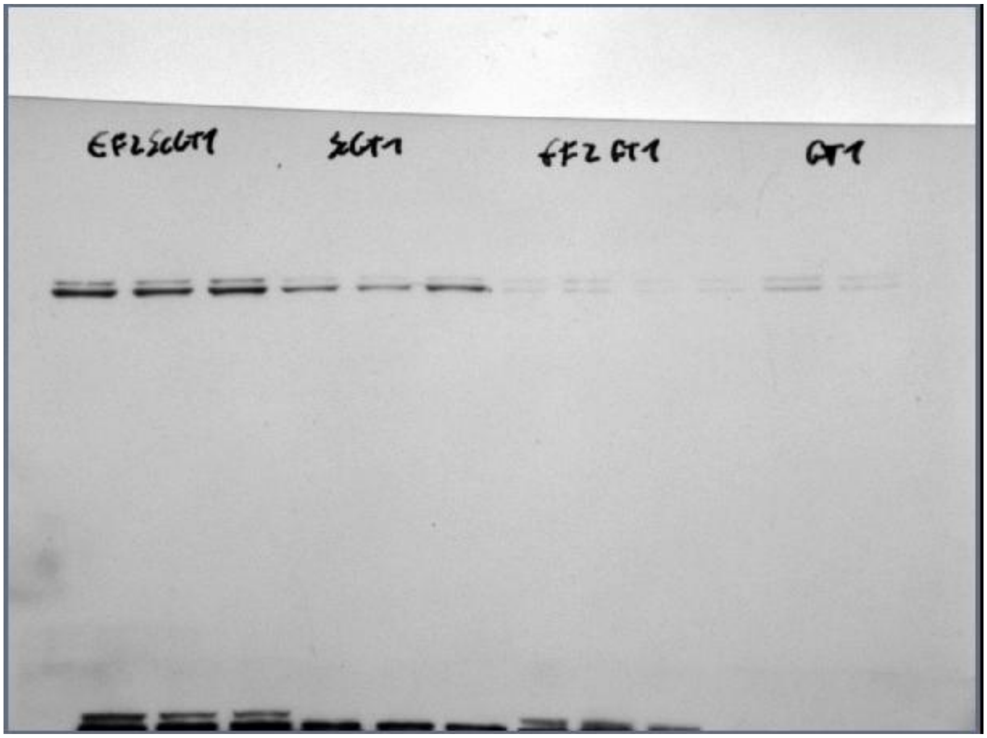

Total ERK blot in GT1 and ScGT1 treated with EB8

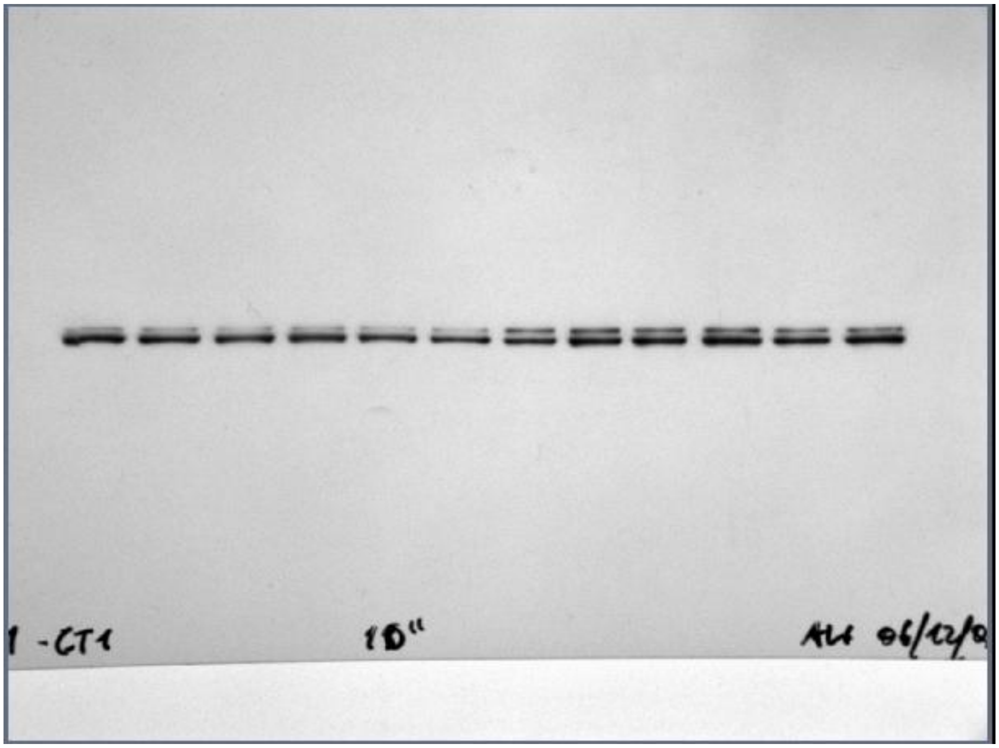

Phospho-ERK blot in GT1 and ScGT1 treated with EB8

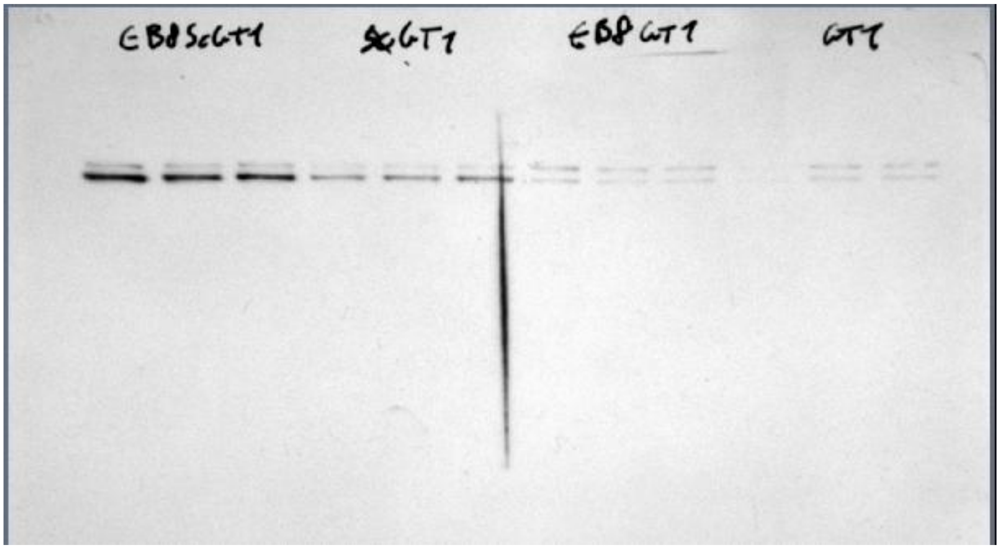

SPR data

DC2

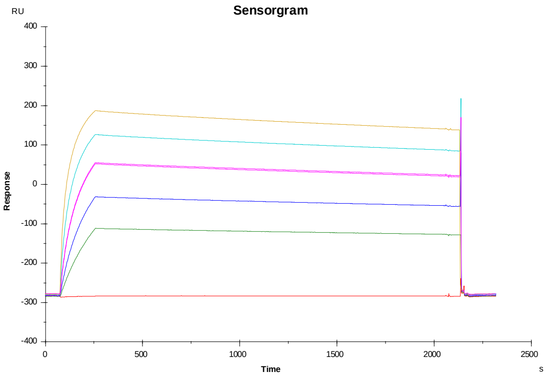

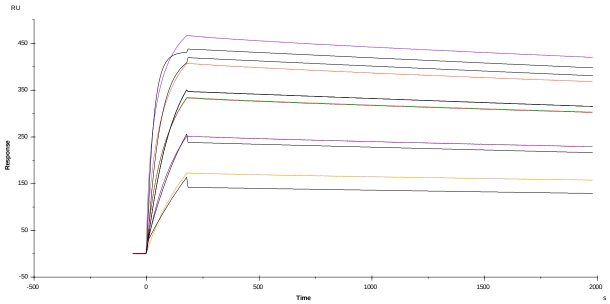

KD=6,144×10^{-10} Chi^{2}=322 (73,3 %)
Rmax=439,3

DE10

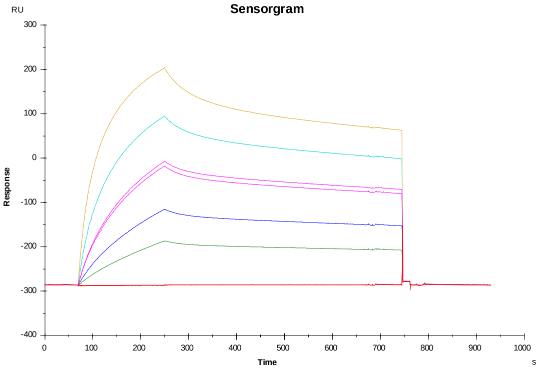

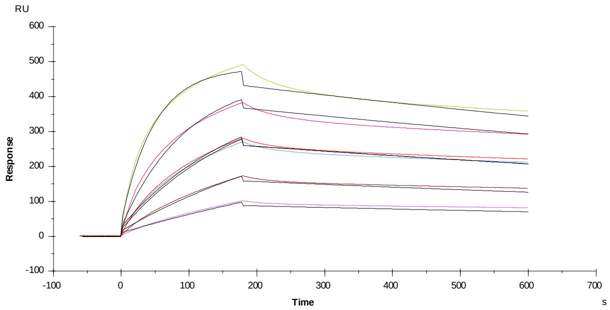

KD=1,071×10^{-8} Chi^{2}=70,5 (15,5 %)
Rmax=454,4

EB8

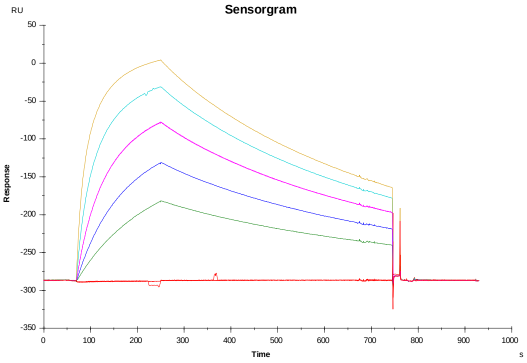

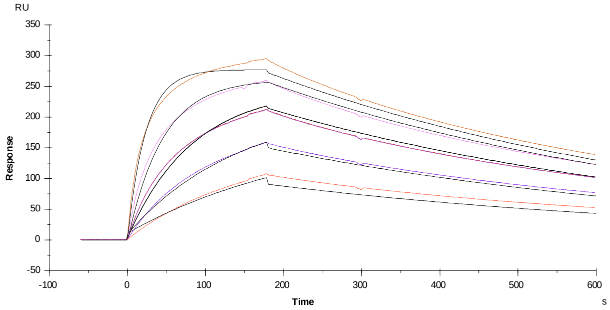

KD=1,714×10^{-8} Chi^{2}=40,9 (48,6 %)
Rmax=284,2

EF2

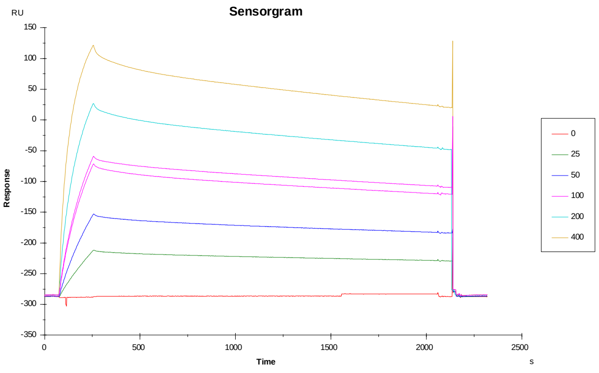

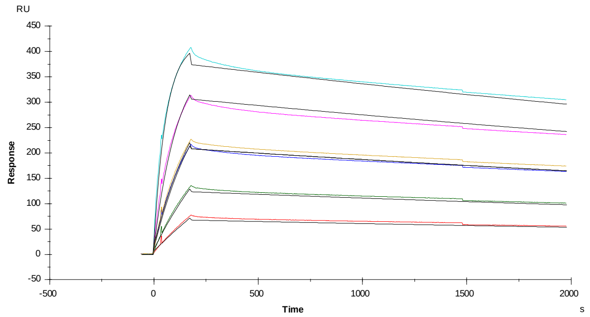

KD=3,084×10^{-9} Chi^{2}=39.2 (9,9 %)
Rmax=395,7

MTT data

DC2 GT1	NC		DC2 ScGT1	NC
0.386	0.012		0.13	0.003
0.437	0.014		0.14	0
0.482	0.017		0.168	0.003
0.452	0.013		0.116	0.002
0.419	0.013		0.142	0.005
EF2 GT1	NC		EF2 ScGT1	NC
0.388	0.013		0.126	0.001
0.42	0.015		0.147	0.002
0.431	0.012		0.141	0.003
0.469	0.015		0.141	0
0.446	0.015		0.112	0
DE10 GT1	NC		DE10 ScGT1	NC
0.469	0.014		0.12	0
0.447	0.014		0.153	0
0.438	0.014		0.187	0.003
0.471	0.016		0.138	0.001
0.451	0.013		0.151	0.004
EB8 GT1	NC		EB8 ScGT1	NC
0.437	0.014		0.113	0.003
0.404	0.013		0.128	0
0.402	0.012		0.108	0
0.432	0.013		0.157	0.002
0.354	0.011		0.126	0.006
GT1	NC		ScGT1	NC
0.373	0.012		0.139	0.002
0.443	0.014		0.153	0.007
0.385	0.013		0.147	0.004
0.397	0.014		0.147	0.008
0.407	0.013		0.118	0.004

Blot replicates

ScGT1 cells treated with EF2

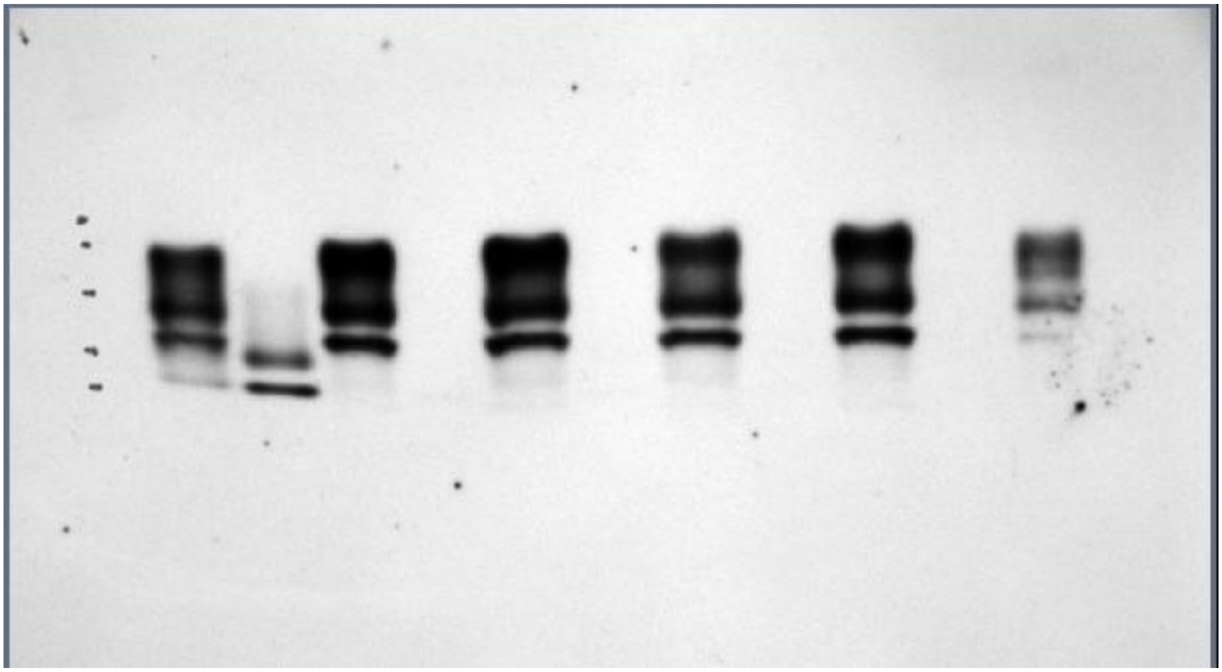

ScGT1 cells treated with DE10

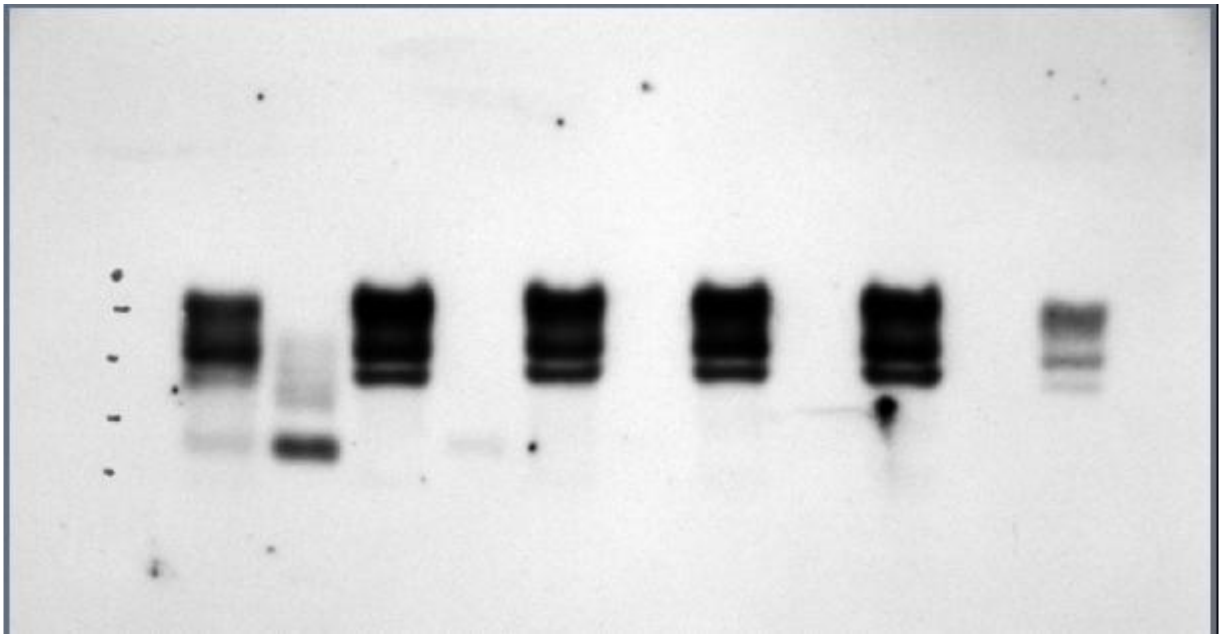

ScGT1 cells treated with EB8

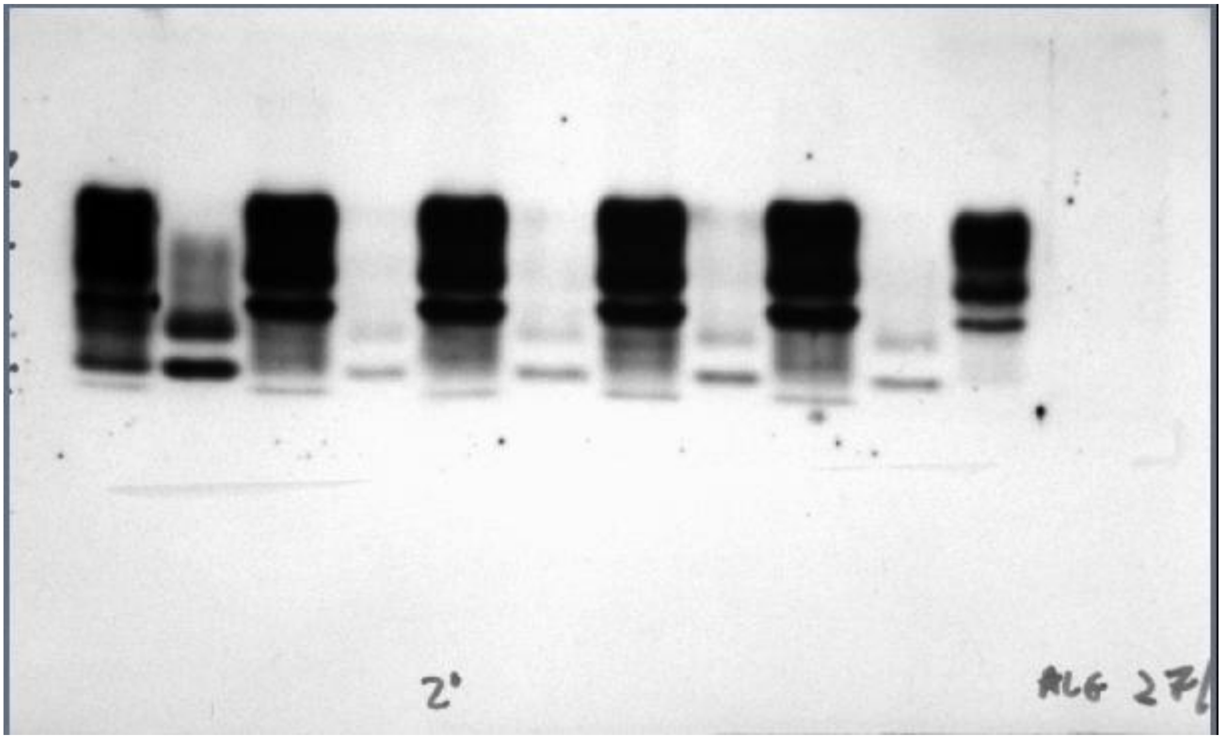

ScGT1 cells treated with DC2

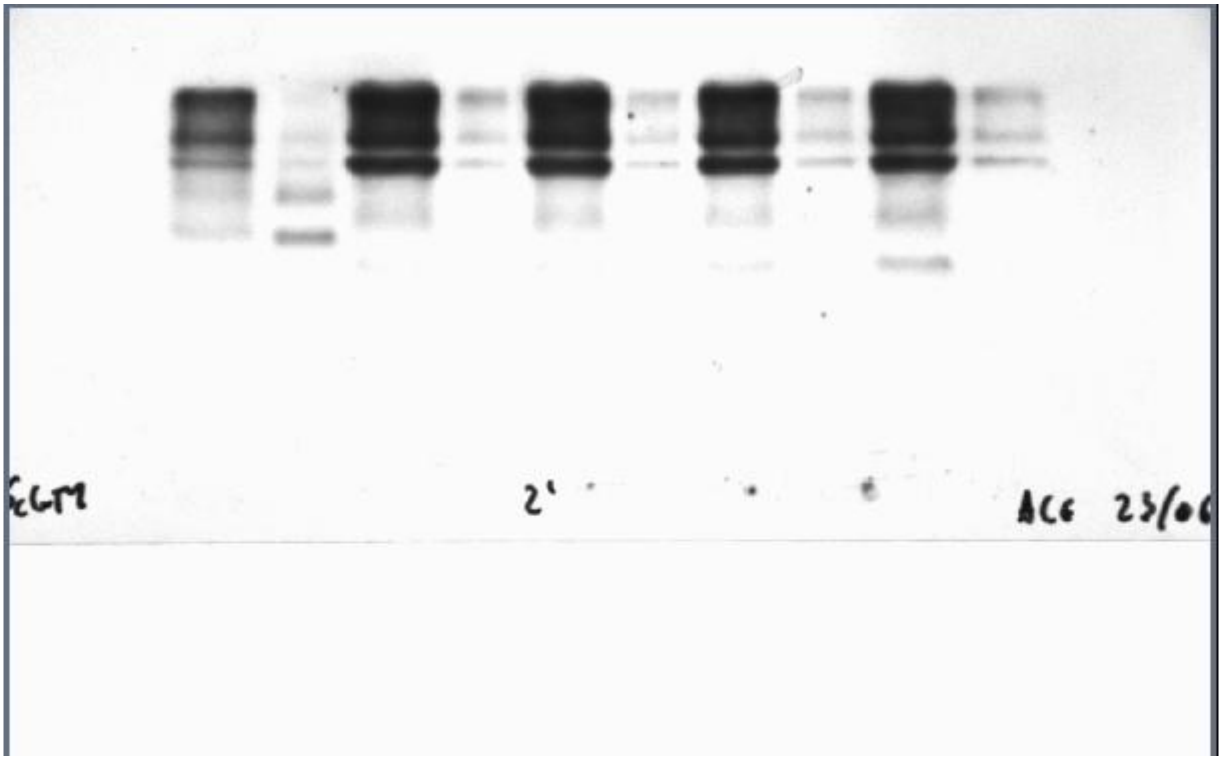

Supplement: Supplemental Information 4 — Raw data for immunoblots, ELISA assays, MTT assays, immunofluorescence and Surface Plasmon Resonance (SPR) experiments. [file peerj-03-811-s004.pdf]
